# Clusters of high abundance of plants detected from local indicators of spatial association (LISA) in a semi-deciduous tropical forest

**DOI:** 10.1371/journal.pone.0208780

**Published:** 2018-12-13

**Authors:** José Ramón Martínez Batlle, Yntze van der Hoek

**Affiliations:** 1 Universidad Autónoma de Santo Domingo, Facultad de Ciencias, Ciudad Universitaria, Santo Domingo D.N., Dominican Republic; 2 The Dian Fossey Gorilla Fund International, Musanze, Rwanda; University of Victoria, CANADA

## Abstract

Plants are rarely randomly distributed across communities, and patchiness is a common spatial pattern in most tropical forests. Clusters of high density of plant individuals are related to internal and external forces, as well as to historical events. The detection of aggregated patterns of plant individuals allows for a better understanding of the internal and external factors that guide the distribution of species. The aim of this research was to detect and characterize clusters of high abundance of plants and species richness in semi-deciduous forests in the Dominican Republic. For this, we collected vegetation data from 575 quadrats in 23 transects (2300 m^2^ in total) within the Ocoa river basin. Using local Moran’s *I* statistics, we isolated 18 quadrats of high density of individuals. We show that density of individuals can be 2.5 times larger on average than in non-aggregated quadrats, and can reach higher values for shrubs species as well as for palms and vines species. In addition, we found that shrub species are the most abundant group in aggregated quadrats, and density of tree species is significantly smaller than that of shrub species. High density quadrats are predominantly occupied by shrubs, palms and vines, following patterns of species composition and lithology. Detecting clusters of high density of individuals could help in the efficient assessment of richness in semi-deciduous tropical forests, and may support new conservation practices for this valuable but threatened ecosystem.

## Introduction

Determining spatial patterns of species assemblages is key to understanding the mechanisms that control species distributions. Spatial patterns in the distribution of communities can be shaped by internal (e.g., population dynamics) and external (e.g., environmental characteristics) deterministic forces, as well as current and historical stochastic events [1]. Analyses of individual-based tree data often focus on quantifying the variation in tropical forest species related to abundance or on evaluating spatial patterns in tropical trees populations. Randomly distributed species are rare across a community [2, 3], and patchiness is found at all spatial scales [1]. For example, tree species are shown to be non-randomly aggregated in tropical forests [3], and lianas have been found to be aggregated due to clonal stem recruitment in treefall gaps [4]. Importantly, growing populations should be more aggregated than shrinking populations, and change in aggregation may lag behind the change in abundance, holding information about population dynamics as a “memory” [5].

Analyses of spatial vegetation patterns carry essential information on the level of spatial dependence of sampling units (e.g., individual plants), an important confounding factor to consider in many ecological data analyses [1, 6, 7]. However, spatial patterns themselves might also be of interest, as they are the key to understanding both the past ecological processes that formed patterns as well as potential future developments in ecosystem function or stability (e.g., due to climate change [8] or transition shifts following catastrophes [9]). A better understanding of the factors that govern pattern formation allows us to transform studies of spatial patterns to predictive studies, that can inform a range of conservation or management efforts, from forest fire management [10] to erosion and runoff control [11].

Analysis of spatial patterns comprises two families of methods: point pattern analysis and surface pattern analysis [1]. The first evaluates distribution through space of individual objects, and the latter deals with spatially continuous variables. In this study, we focus on point pattern analysis of individuals of plants, using data collected in transects from semi-deciduous tropical forest of the Dominican Republic, to detect and characterize spatially aggregated patterns of woody species using local Moran’s *I*, a local indicator of spatial association (LISA) proposed by Anselin [12]. We address whether Moran’s *I* as an indicator to detect spatial aggregation at quadrat-level. We explore which species and growth-forms are common in spatially aggregated units, and whether they are similar to those in non-aggregated ones. Finally, we assess whether there is an association between lithology and species composition in aggregated quadrats.

We are the first to study spatial vegetation patterns in semi-deciduous forests in the Dominican Republic, forests that are known to be highly threatened by local human disturbances, such as burning for charcoal production [13], as well as larger-scale effects stemming from climate change (seen also in other regions of Central America and the Caribbean [14]). Little semi-deciduous forest remains in the Dominican Republic (*ca*. 5% of the entire country [15]), and many associated species are under threat of extinction, including the endemic *Acacia skleroxyla*, *Coccothrinax argentea*, *Ottoschulzia domingensis* and *Coccoloba buchii*, and the endangered *Swietenia mahagoni*, *Amyris elemifera* and *Guaiacum officinale* [16]. Our study provides information for current management of remaining forest, ecological restoration efforts and predictions of future developments.

## Materials and methods

We collected vegetation data with the aim to detect and characterize clusters of high plant abundance through spatial analyses. For this, we isolated clusters of high density of individuals within our sample with local Moran’s *I* statistics. Subsequently, we assessed the proportion of individuals by growth habit in clusters compared to the rest of the sample. Finally, we ordinated clusters to assess potential association with lithology.

**Fig 1:**
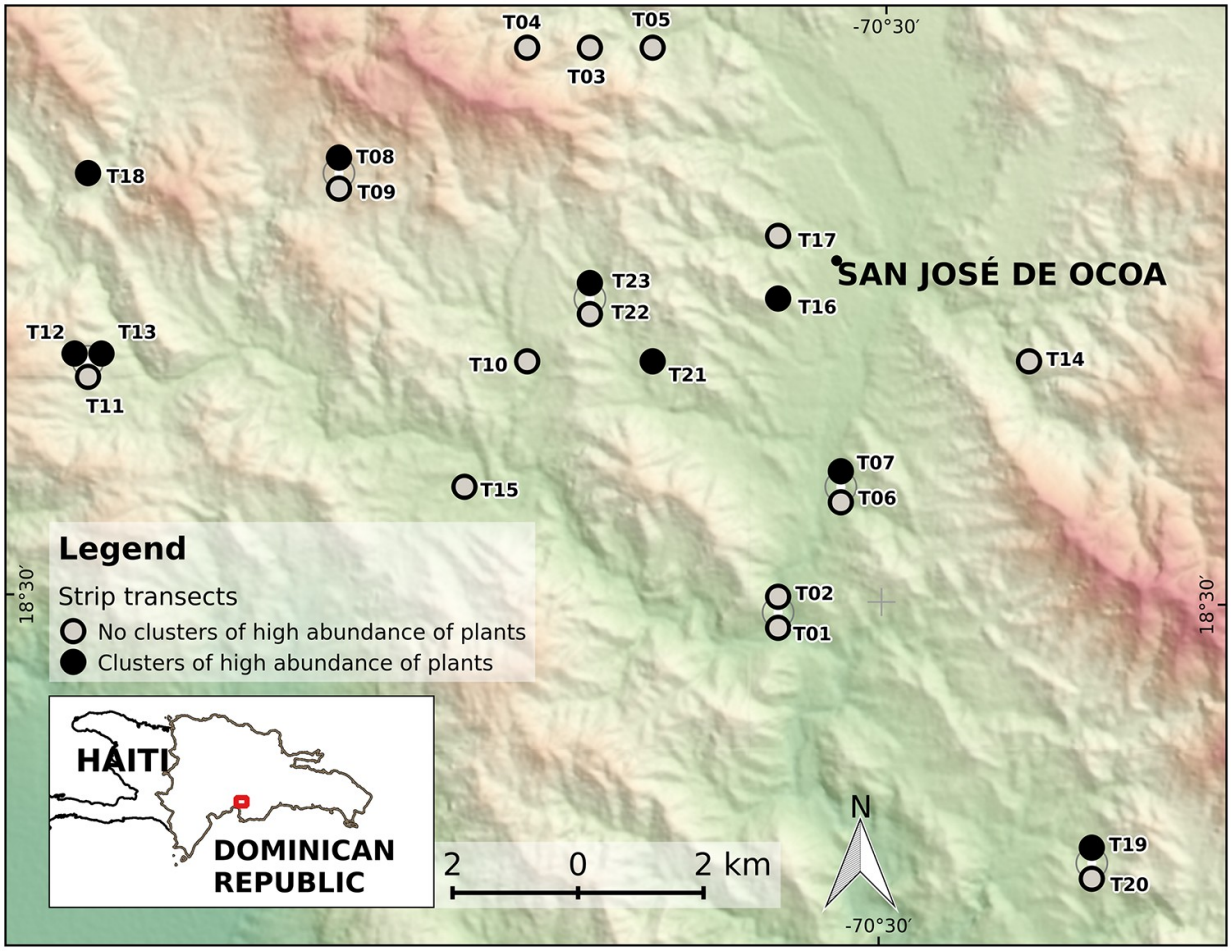
Location map of 23 transects (circles), showing the town of San José de Ocoa. The overlapping points are depicted slightly displaced from their actual positions to ensure that all become visible. The circles filled black depict transects in which clusters of high abundance of plants were detected. The layout of Transect 11 (T11) is shown as an example in Fig 2. The background is a color shaded-relief view (red-white is highland, green is lowland) based on a 30-m SRTM DEM (Ref: NASA LP DAAC, 2000. SRTM 1 Arc-Second Global. https://earthexplorer.usgs.gov/. Published September 2014). The bottom-left inset shows the area in the context of the Dominican Republic.

**Fig 2:**
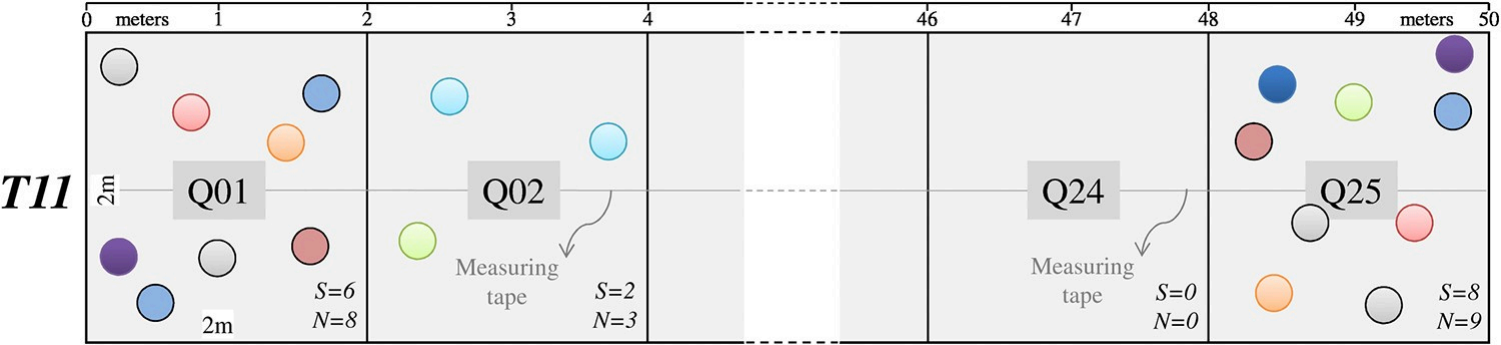
Layout of Transect 11 (T11) showing quadrats sized 2 m × 2 m (4 m^2^) (gap at the center). Circles are individuals, each color represents a species. Q1, Q2,…, Q24, Q25 codes inside grey shaded squares, are quadrats ID. *S* and *N* account for the species number and the total abundance in each quadrat, respectively. Tick marks on the top margin indicate the transect length in meters.

During two field seasons, fall 2013 and summer/fall 2014, we collected data using an adapted version of the Gentry transect procotol [17]. In the original version, this consists of at least ten 50 m × 2 m (100 m^2^) randomly located transects to sum one tenth hectare, “recording lianas and all trees and large shrubs over 2.5 cm dbh” [17]. In our adaptation, based on Cámara & Díaz del Olmo [18], we established 23 transects within the Ocoa river basin of the Dominican Republic (18° 31’ N, 70° 33’ W) (Fig 1), dividing each into 25 evenly placed quadrats sized 2 m × 2 m (4 m^2^, see Fig 2), to reach 575 quadrats that sum 2300 m^2^ of area sampled. In all quadrats, we recorded a total of 2158 individuals of 172 species of woody trees, woody vines or lianas, palms and cacti, 1.5 m height or greater, within “semideciduous forest of *Swietenia–Coccoloba*” [19]. An expert botanist from the National Botanical Garden of Santo Domingo aided us with the identification of the species.

We used the criteria based on height and branching for differentiating between trees and shrubs [20, 21]. We classified as trees the perennial woody plant species at least 6 m in height at maturity and having an erect trunk. Likewise, we classified as shrubs those woody species that remain smaller than 6 m and ramified from the base. Finally, we labeled as vines or lianas those long-stemmed woody plants rooted at the soil which climb to the canopy using trees and shrubs.

All fieldwork was conducted under a permit issued by the Ministry of Environment and Natural Resources. In cases where access to forest inside private land was necessary, oral on-site permission was requested.

Desired locations of transects were obtained using a random points generator tool in QGIS [22]. We restricted each point to fall within a range of 400-800 m above sea level (based on a reclassified ASTER DEM [23]) and under a forest cover of 35% or greater (estimated from a NDVI reclassified Landsat image). The sampling method also restricted that all common rock and landform types found in the basin were represented in our samples. Due to access difficulties and inherent risks of field work, locations of certain points were adjusted to more feasible positions, keeping resemblance with original features.

Rock types and landforms were homogeneous within each transect. Rock identification was accomplished with field recognition and consultation of geological maps [24]. Landforms were observed in the field, and confirmed with aerial photography [25].

We digitized data in LIBREOFFICE CALC worksheets [26]. In order to avoid the use of unaccepted names, duplicates and synonyms, we cleaned our data consulting international databases [27, 28] and reviewing previous research [29, 30]. With the support from an expert botanist, we also classified species by their growth habit in one of tree, shrub, liana/vine, palm or cactus. We included the full names of the plant species used in this paper in S1 Table.

All analyses were conducted in R [31]. We used packages spdep [32], sp [33, 34], spatstat [35–37] and gstat [38, 39] for spatial analysis of abundance across quadrats. For data management, we used packages reshape2, dplyr and tidyr [40–42], and for diversity analyses, packages vegan [43] and BiodiversityR [44].

We used “number of individuals of all species pooled per square meter for each quadrat” (hereafter “density of individuals”) as spatially analyzed variable, calculated according to *dN = N/4*, where *N* is number of individuals per quadrat. We estimated spatial association for *dN* from local Moran’s *I* statistic, as proposed by Anselin [12], a class of indicators known as “local indicators of spatial association/autocorrelation” (hereafter “LISA”) [45], with the following equation:

$$\begin{array}{c} I_{i}=\frac{\left(x_{i}-\bar{X}\right)}{S_{i}^{2}}\sum_{j=1,j\neq i}^{n}w_{ij}\left(x_{j}-\bar{X}\right) \end{array}$$ (1)

where *I_i_* is local Moran’s *I* for entity *i*, *x_i_* is the value for the variable of interest in *i*, $\bar{X}$ is the mean of the variable, *n* is the number of spatial entities, *w_ij_* is the spatial weight of the link between entities *i* and *j*, and $S_{i}^{2}$ is:

$$S_{i}^{2}=\frac{\sum_{j=1,j\neq i}^{n}{\left(x_{j}-\bar{X}\right)}^{2}}{n-1}$$ (2)

Spatial weights *w_ij_* are row standardized or “*W* style” (e.g. *w*_*1-1*_+…+*w*_*1-25*_ sums 1), and derived from first-order contiguous neighbours, so each quadrat has two neighbours except for those at the endpoints of the transect which have only one neighbour. Thus, the quadrats directly neighbouring a central quadrat are each given a weight of 0.5, while the rest are given zero weight. This neighbouring and weighting style is suitable for a comparative interpretation of *I* among transects, since each quadrat has both the same number of neighbours and the same weight values are given to its neighbours.

We also ran “local Moran’s *I* tests with Bonferroni correction for p-values”, under the null hypothesis of “no spatial autocorrelation”, assuming a significance level α = 0.05. In order to avoid false positive results in the endpoint quadrats due to the edge effect, we assumed a lower significance level α = 0.01, and we also checked whether the neighbouring quadrats showed high density of individuals.

Moran’s *I* gives a formal indication of the degree of linear association between a vector of observed values $x-\bar{X}$, and a weighted average of the neighbouring values $W(x-\bar{X})$, or spatial lag [46]. Since these values are deviations from their mean, Moran’s *I* is equivalent to a slope coefficient in a regression of $W(x-\bar{X})$ on $x-\bar{X}$. An easy way to visualize this linear association is a scatterplot of $W(x-\bar{X})$ against $x-\bar{X}$, known as “Moran scatterplot” [46, 47]. We generated Moran scatterplots for each transect, considering quadrats within them as entities to plot.

In addition, we plotted *LISA cluster maps*. A LISA cluster map classifies each entity by the type of significant association between observed values and their respective lagged ones [48], in four different classes: *High-High*, *Low-Low*, *High-Low* and *Low-High*. The first two, *High-High* and *Low-Low*, are so called “clusters”, which respectively means “high surrounded by high” and “low surrounded by low”. The other two are “spatial outliers”, where *High-Low* means “high surrounded by low”, and *Low-High* are “low surrounded by high”; these classes were absent in our data. A fifth class called “not significant”, encompasses all entities in which there were no significant association, and are supposed to have random values of the variable; this class dominated our LISA cluster map. In this research we focused only in *HH* clusters of “density of individuals”; *LL* clusters and “not significant” are considered “the rest of the sample”.

After identifying local spatial *HH* clusters, we analyzed their abundance and richness with respect to the rest of the sample. We also compared relative abundance of plants by growth habit between clusters and the rest of the sample, as well as species composition with rank-abundance tables [49, 50].

We generated two ordination diagrams in order to assess groups of *HH* clusters in relation to species composition and lithology. We performed a Principal Coordinate Analysis (PCoA) using a Bray-Curtis distance matrix, which we calculated from raw abundance data, and we also performed a Principal Component Analysis (PCA) using Hellinger-transformed abundances [1, 51]. We used the graphical outcomes from both methods to explore the community ordination of the *HH* clusters.

In order to determine the separation between groups, we tested several hierarchical clustering methods using a chord distance matrix. Afterwards we chose the dendrogram with the highest cophenetic correlation value, which in our sample was the one generated with the unweighted pair-group method using arithmetic averages, hereafter UPGMA. Finally, we identified groups using the multiscale bootstrap resampling algorithm from the pvclust R package [52], which we applied to the UPGMA dendrogram.

## Results and discussion

**Fig 3:**
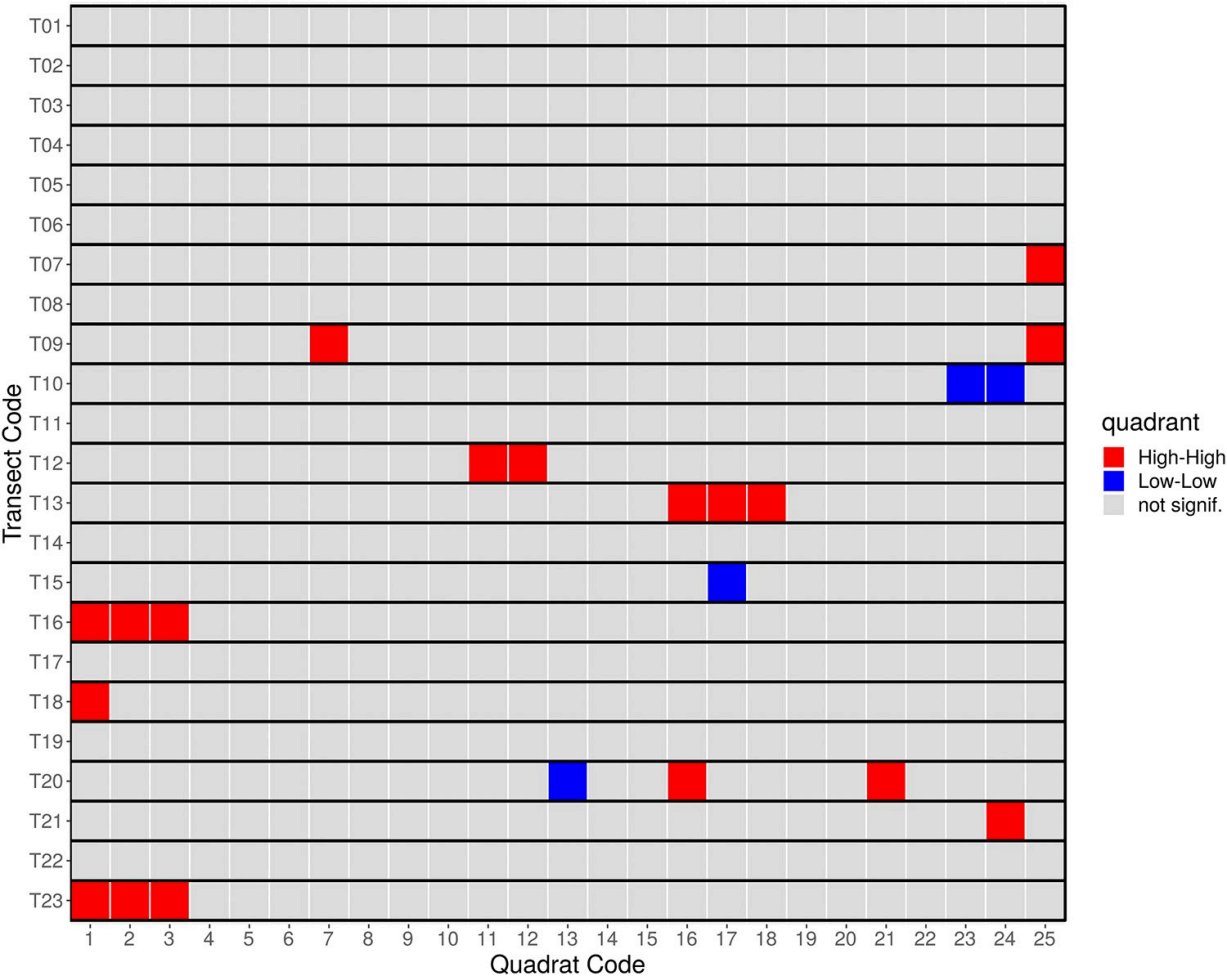
Panel of Moran scatterplots for lagged densities of individuals (*ldN*) against non lagged respectives (*dN*). Each scatterplot represents a transect and dots represent quadrats; those spatially associated with high values surrounded by high (so called *HH* clusters) are coloured in red. A regression grey line is also plotted. *HH* clusters quadrats are coloured in red, of which only 16 are visible, 2 less than the actual number, because overlap of dots (T16Q01/T16Q03 and T20Q16/T20Q21).

We calculated local Moran’s *I* for density of individuals of all growth habit (*dN*) in the 575 quadrats. Twenty two showed significant spatial associations of *dN*, 18 of which can be considered *HH* clusters (see Table 1 and Fig 3).

**Table 1:** Quadrats with significant association (to α = 0.05) between high observed values of density of individuals and high lagged respectives ones, so called “spatial clusters of type *High-High*” or *HH* clusters.

Transect	Quadrat	*N*	*dN*	Lagged *dN*	*I*_*i*_	Pr(z>0)
T07	Q25	5	1.25	3.00	2.42	<0.01
T09	Q07	7	1.75	1.75	1.78	0.010
	Q25	8	2.00	1.75	2.47	<0.01
T12	Q11	13	3.25	2.12	2.02	<0.01
	Q12	14	3.50	2.00	1.94	<0.01
T13	Q16	12	3.00	2.50	1.75	0.01
	Q17	10	2.50	3.25	2.10	<0.01
	Q18	14	3.50	2.62	2.86	<0.01
T16	Q01	12	3.00	2.00	2.02	0.03
	Q02	8	2.00	3.00	2.02	<0.01
	Q03	12	3.00	2.00	2.02	<0.01
T18	Q01	4	1.00	1.00	2.91	<0.01
T20	Q16	10	2.50	2.12	1.67	0.02
	Q21	10	2.50	2.12	1.67	0.02
T21	Q24	9	2.25	1.12	1.46	0.04
T23	Q01	8	2.00	1.75	6.46	<0.01
	Q02	7	1.75	1.62	4.72	<0.01
	Q03	5	1.25	1.12	1.34	0.048

*N*, number of individuals; *dN*, density of individuals; lagged *dN*, respective weighted values of *dN*; *I_i_*, local Moran’s *I* statistic; Pr(z>0), p-value resulting from testings under the null hypothesis of no spatial association.

Most of the quadrats are not spatially associated with their neighbours in terms of density of individuals, so spatial clusters are rare in this type of forest. Also we noticed that there are no spatial outliers, or so called hotspots (*High-Low* class type), which means that there are no quadrats with significant high density values surrounded by low density ones. This likely indicates that in this region we find no abrupt spatial changes in factors driving spatial variation in plant distribution, but rather gradual transitions among environmental variables [53].

**Fig 4:**
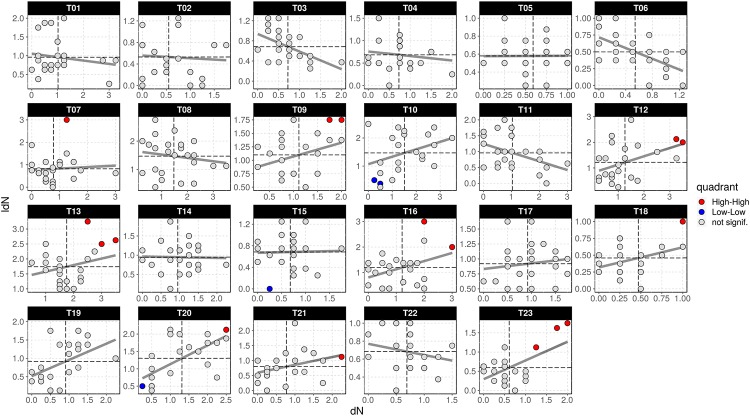
LISA cluster map showing 575 quadrats (from 23 transects), each classified as clusters *High-High* (red), *Low-Low* (blue) and “not significant” (grey), regarding to their density of individuals. Even though each row represents one transect, the transects were not placed adjacent to one another. See text for details.

LISA maps (Fig 4) show that *HH* clusters may contain two or more contiguous quadrats, which is a expected behavior, as shown by transects T13, T16 and T23. Clusters may also occur as isolated entities, with one or more associated quadrats per transect, as for example in T07, T09 or T21, to cite only three cases. The high number of quadrats with no significant association, remarks the infrequent aggregation of abundance in this forest type.

As expected, *HH* clusters concentrate high values of richness and abundance in a small area. These 18 quadrats account for *ca*. 36% of the overall species richness (61 species) and 8% of abundance (168 individuals), but only represent 3% (18 × 4 m^2^ = 72 m^2^) of the sample area. A comparison between *HH* clusters, the entire sample and the rest of quadrats, is presented in Table 2.

**Table 2:** Comparison of traits between entire sample, *High-High* clusters and the rest of the sample, in terms of richness, abundance, area, and percentage of individuals and density by growth habit. See text for details.

Trait	Entire sample	*HH* clusters	Rest of the sample
**General**			
Number of species	172	61	167
Number of individuals	2158	168	1990
Number Quadrats	575	18	557
Area, in m^2^	2300	72	2228
**Growth habit, in % of individuals**			
Trees	50.56	34.52	52.35
Shrubs	38.14	42.26	37.27
Palms	5.00	10.72	4.60
Vines and cacti	6.3	12.50	5.78
**Density, in individuals/m^2^ (mean±standard error)**			
Trees	0.474±0.018	0.806±0.138	0.464±0.018
Shrubs	0.358±0.019	0.986±0.180	0.338±0.018
Palms	0.047±0.007	0.250±0.114	0.040±0.006
Vines and cacti	0.020±0.002	0.097±0.029	0.017±0.002
Undifferentiated (pooled)	0.156±0.006	0.389±0.056	0.149±0.006

Percentages of individuals per quadrat classified by growth habit, show that shrubs, vines/cacti and palms are proportionally more abundant in *HH* clusters than in the rest of the sample; conversely, average percentage of trees decrease in *HH* clusters with respect to the rest of the sample.

**Fig 5:**
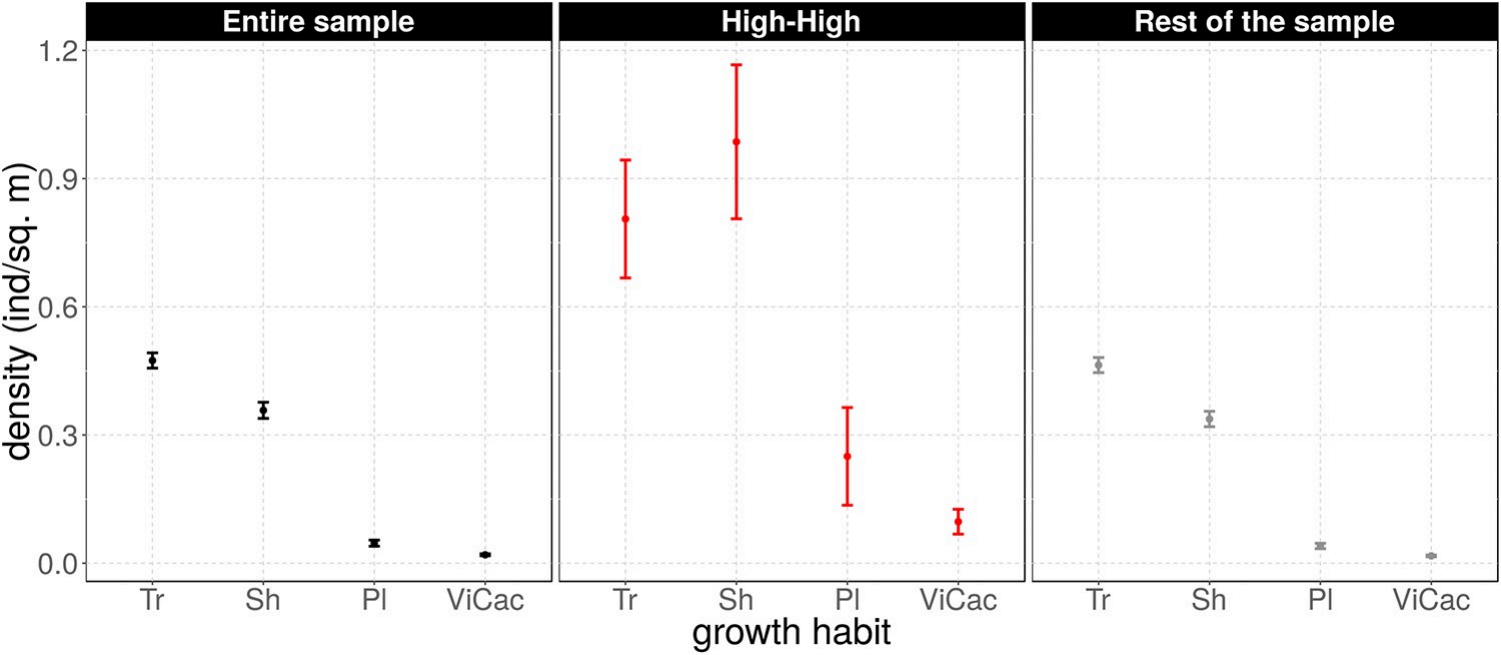
Density of individuals vs. their species growth habit type, for three types grouping criteria of the quadrats according to their spatial association of individuals. (A) Entire sample, or undifferentiated spatial association; (B) *High-High* clusters; (C) Rest of the sample, or non-aggregated pattern of individuals. Legend: *Tr*, trees; *Sh*, shrubs; *Pl*, palms; *ViCac*, vines and cacti. See text for details.

Densities values are also significantly larger (almost 2.5 times on average) in *HH* clusters than in the rest (*t* = *4.2*, *df* = *110*, *p* ≪ *0.01*). Densities of shrubs (*ca*. 1 individuals/m^2^), palms (0.25 individuals/m^2^) and vines (*ca*. 0.1 individuals/m^2^) in *HH* clusters are respectively 2.9, 6.2, and 5.7 times higher than in other quadrats in our sample. Although tree density is *ca*. 0.8 individuals per square meter in *HH* clusters, shrub density is 1.25 times larger, which is a reversed pattern compared to the rest of the sample (see Fig 5), where density of trees is higher.

We analyzed the rank-abundance tables of the 17 most abundant species present in at least 3 quadrats for both the *HH* clusters quadrats and the rest of the sample (see Table 3). *HH* clusters (left half of the table) have 11 out of 17 species which their growth habits are shrubs, palms and vines.

**Table 3:** Rank-abundance tables of the 17th most abundant species present in at least 3 quadrats for both *HH* clusters (left half of the table) and the rest of the sample (right half of the table). Each group has 17 species. See text for details.

*HH* clusters (pooled)			Rest of the sample (pooled)	
Species	Prop.	Rank	Species	Prop.
*C. argentea*	15.90	1	*C. diversifolia*	6.40
*C. haematomma*	12.10	2	*R. aculeata*	5.40
*R. aculeata*	9.30	3	*C. argentea*	4.7
*C. diversifolia*	7.50	4	*B. simaruba*	4.50
*E. foetida*	6.50	5	*E. foetida*	4.40
*B. simaruba*	5.60	6	*S. sessiliflora*	4.40
*C. buchii*	5.60	7	*A. skleroxyla*	4.30
*E. odorata*	5.60	8	*C. oliviforme*	3.20
*S. dodecandra*	5.60	9	*L. leucocephala*	2.90
*E. paniculata*	4.70	10	*S. mahagoni*	2.80
*A. gummifera*	3.70	11	*C. buchii*	2.60
*C. oliviforme*	3.70	12	*S. dodecandra*	2.40
*C. alba*	2.80	13	*V. macracantha*	2.30
*C. dodonaea*	2.80	14	*N. coriacea*	2.30
*J. fluminense*	2.80	15	*P. pentandra*	2.30
*P. sulcata*	2.80	16	*T. pallida*	2.0
*T. berteroi*	2.80	17	*G. mollis*	1.80

*Species*, Latin binomial abbreviated (see S1 Table for full names). *Prop*. stands for proportion of individuals in the group (proportional abundance in %). Color shading stands for growth habit of each species: tree, shrub, palm, vine

Specifically, only three of the ten most common plant species in *HH* clusters were trees. The other seven were an endemic palm *Coccothrinax argentea* and six shrub species (*Eugenia foetida*, *Calliandra haematomma*, *Randia aculeata*, *Coccoloba buchii*, *Eugenia odorata* and *Samyda dodecandra*). These results confirm that shrubs, palms, and vines are relevant to form aggregated patterns among dry forest vegetation.

Of the 17 most abundant species present in at least 3 quadrats in the rest of the sample (non-*HH* clusters, right half of Table 3), 10 are trees, 6 are shrubs and 1 is a palm. The tree *Coccoloba diversifolia*, is the most common species of this type of quadrats, and represents almost 6% of their abundance.

We highlight the presence of *Leucaena leucocephala* (Leguminosae), one of the world’s most invasive plants species which was introduced in the Dominican Republic in the last century. It is ranks 9th most abundant in non-*HH* clusters, and was present in 41 out of 557 quadrats, which confirms the highly invasive nature of this species [54–56]. This species was also present in two quadrats of the *HH* clusters, so it may contribute slightly to the observed aggregation pattern.

Rank-abundance tables also show that some common trees in the rest of the sample, such as *Savia sessiliflora*, *Acacia skleroxyla*, *Leucaena leucocephala*, *Swietenia mahagoni*, *Vachellia macracantha*, *Nectandra coriacea* and *Trichilia pallida*, are rare or absent in *HH* clusters. Evidence suggests that these species appear isolated in the forest, or at least with few or non shrubs/palms/vines in the direct vicinity (<2m).

**Fig 6:**
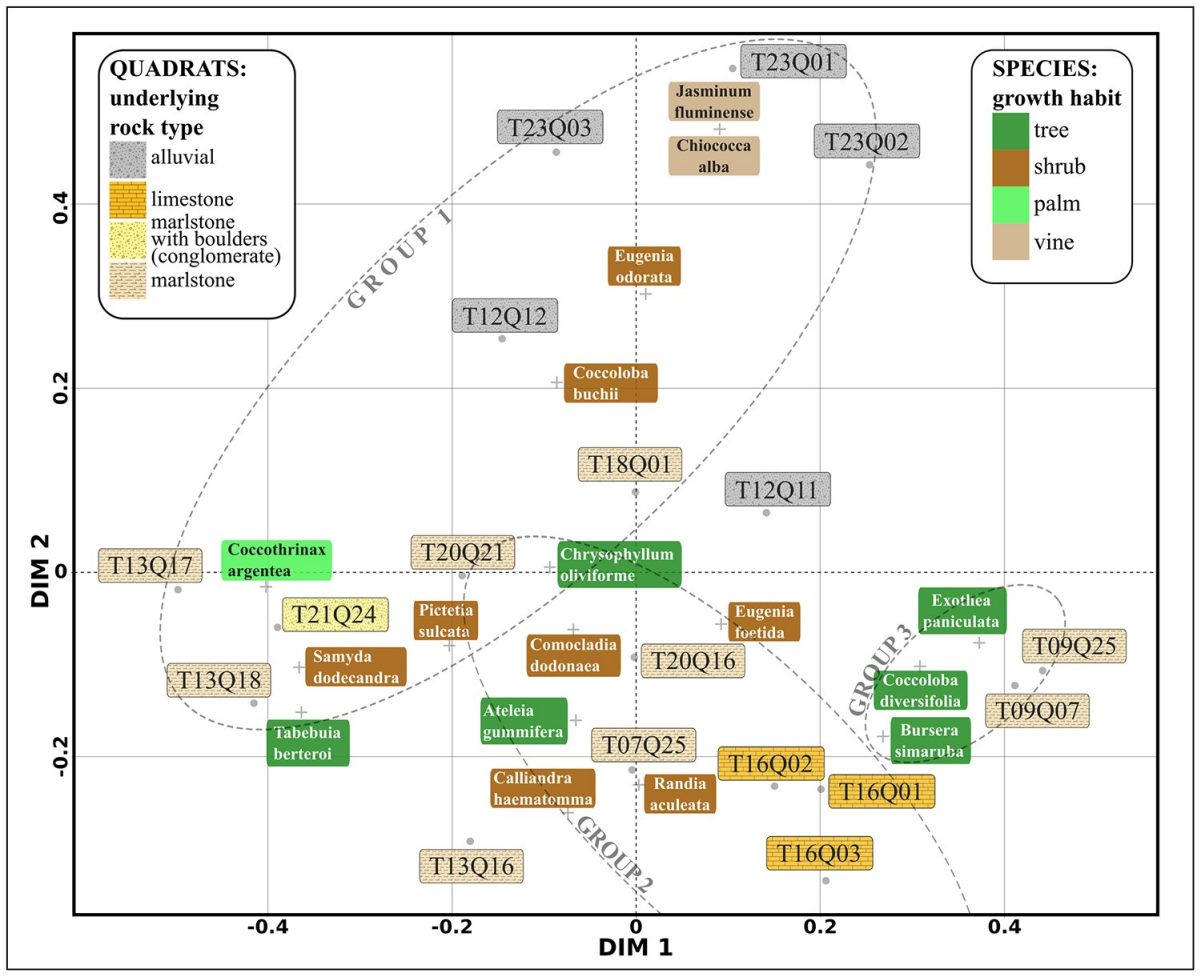
Ordination plot from Principal Coordinate Analysis (PCoA) of the 18 *HH* clusters quadrats, showing only the 17th most abundant species (from 61) present in at least 3 quadrats. Names boxes are coloured according to growth habit in the case of species, and underlying rock type for quadrats. See text for details.

**Fig 7:**
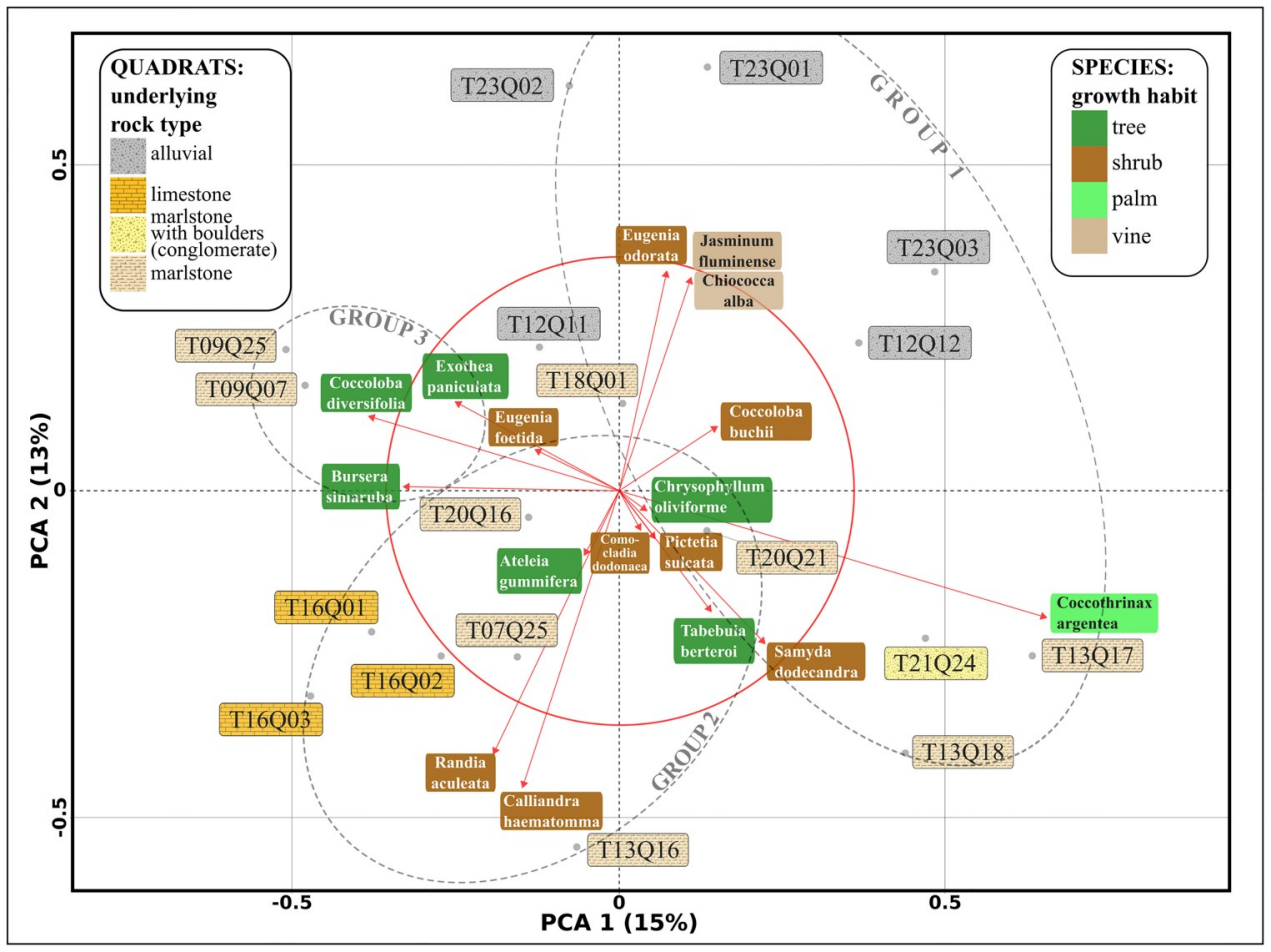
Ordination plot from Principal Component Analysis (PCA) of the 18 *HH* clusters quadrats, showing only the 17th most abundant species (from 61) present in at least 3 quadrats. Names boxes are coloured according to growth habit in the case of species, and underlying rock type for quadrats. See text for details.

**Fig 8:**
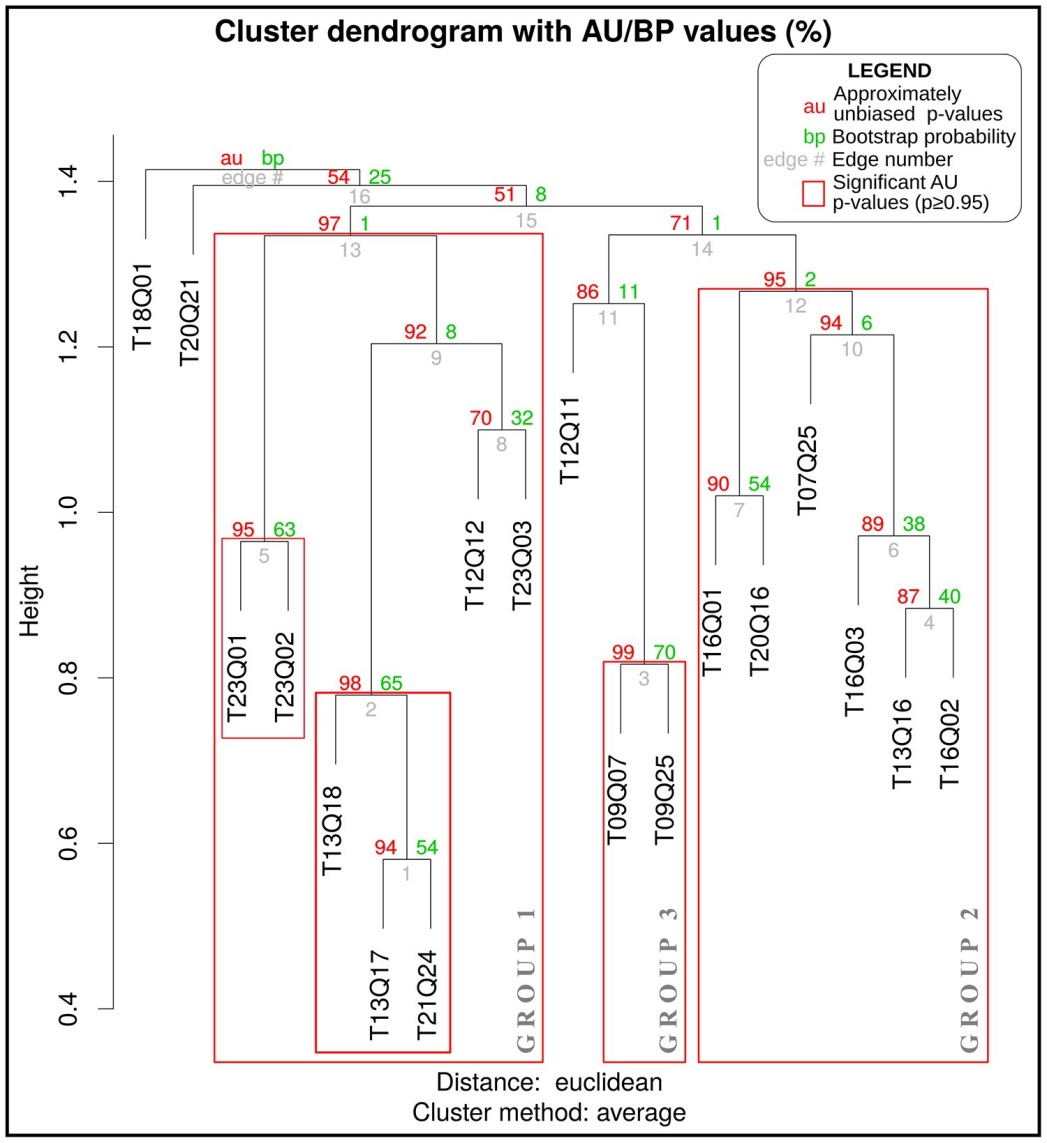
Cluster dendrogram of the 18 *HH* clusters quadrats using the unweighted pair-group method with arithmetic averages (UPGMA), indicating the approximately unbiased p-values (AU) and bootstrap probabilities (BP), generated by the multiscale bootstrap resampling algorithm. See text for details.

We generated ordination diagrams from a PCoA and a PCA in order to assess how groups of *HH* clusters relate to plant species composition and local variation in lithology (see Figs 6 and 7). The results from both methods were consistent in extracting the structure of our data. In addition, the UPGMA cluster dendrogram generated by the multiscale bootstrap resampling algorithm (see Fig 8) suggests that the *HH* clusters can be separated in three classes of quadrats.

We characterize these three classes of quadrats according to their underlying rock type and species composition:

1. Group 1. Quadrats with riparian forest on river bank systems consisting of alluvial deposit, predominantly with *Eugenia odorata*, *Coccoloba buchii*, *Jasminum fluminense* and *Chiococca alba*. Within this group we also found quadrats with forests on low steepness slopes consisting of marlstones, in which tree cover loss was reported between 2001 and 2014, predominantly with *Coccothrinax argentea*, *Samyda dodecandra* and *Tabebuia berteroi*.
2. Group 2. Quadrats with forest on slopes of varied steepness consisting of limestone and marlstone, predominantly with *Calliandra haematomma*, *Randia aculeata* and *Eugenia foetida*
3. Group 3. Quadrats with forest on steep slopes consisting of sandy marlstone, intercalated with sandstone and boulders, predominantly with *Coccoloba diversifolia*, *Exothea paniculata* and *Bursera simaruba*.

Although a previous study indicated the existence of plant distribution clusters in Neotropical dry forests at regional scales [57], we show for the first time that patchiness and clustering in patterns of plant distribution occurs at small, local, scales in dry forests of the Caribbean. This finding has consequences for spatial planning of protected areas in the region [58] (the Caribbean or at least the Dominican Republic), as we are now able to ensure we include locations associated with specific vegetation clusters in protected areas. This, in turn, will allow us to protect a wider range of biodiversity. Moreover, our results provide an important reminder that spatial distribution patterns, especially those of rare species, should be considered in the design of monitoring efforts of dry forests and associated biodiversity [59]. Not considering spatial patchiness may bias population estimates through the inclusion or exclusion of even a few high abundance plots or quadrants. In other words, the detection of clusters of high densities of plants aids efficient monitoring of semi-deciduous tropical forests.

Our results also provide necessary background information to studies of species responses to patchiness in plant distribution patterns, which is especially important for conservation in the context of habitat fragmentation (see e.g., [60, 61]). For example, certain tree species found in our study region were found in relative isolation in the forest, which may have consequences for the ecology and conservation of herbivores or pollinators associated with these species. Moreover, we provide context for follow-up studies on a broad range of topics, such as the effects of the spread of non-native plant species *L. leucocephala* on other organisms [62] and the consequences of observed patchiness on population genetics [63].

The knowledge we provide can also be used to inform reforestation and forest restoration efforts in dry forests in this region [64]. In particular, we can use our understanding of spatial vegetation patterns to identify specific species to be used in reforestation, for example focusing on several of the high abundance shrub species or the endemic palm *Coccothrinax argentea*. Moreover, we are among the first to indicate how species found in tropical dry forests found in Dominican Republic associate with each other, and how their distribution relates to variation in lithology (see Figs 6 and 7). We can now start monitoring spatial and temporal changes in these vegetation compositions, for example to investigate how climate change differentially affects plant species with different traits (e.g., species with different growing habits, as addressed in this study) and consequentially induces changes in community composition [65]. Plus, we can use our knowledge of spatial patterns to test for species-specific associations with particular soil and environmental characteristics, beyond broad associations with lithology. This will allow us to monitor the response of these dry forests to other environmental changes, for example those resulting from agricultural practices and associated nitrogen deposition [66] and provides insights in processes of succession and forest recovery [67, 68]. As such, our study can guide conservation practices in the Dominican Republic and neighboring countries, and provides important baseline information on spatial patterns that explain, or result from, more complex ecosystem dynamics [57, 69, 70].

## Conclusions

We show that spatial analysis patterns assessment, based on local Moran’s *I* statistic and LISA mapping, is suitable to detect concentrations of plants in so called *HH* clusters. In dry forests in the Dominican Republic, density of individuals in these clusters is significantly higher than at other locations. Average individual plant density was up to 2.5 times higher in these clusters than at other random locations in the dry forest. We found that in *HH* clusters, shrub species are the most abundant group, and that the density of tree species is significantly smaller. These densely vegetated quadrats are especially occupied by shrubs, palms and vines, and there is a pattern that is potentially associated with species composition and lithology.

Detecting clusters of high density of individuals could help in the efficient assessment of richness in dry tropical forests, and may support monitoring efforts of the associated biodiversity of these forests. Our findings could be used to inform reforestation and forest restoration efforts in dry forests in the Caribbean region, in particular in identifying specific species to be used in reforestation. Finally, our results may support predictions of how these forests will respond to climate change.

## Supporting information

S1 Table Full names of the plant species mentioned in this paper. (PDF)
