# Supplementary material for: Clusters of high abundance of plants detected from local indicators of spatial association (LISA) in a semi-deciduous tropical forest
Source: PLoS One. 2018 Dec 13;13(12):e0208780. doi: 10.1371/journal.pone.0208780 (PMC6292623; doi:10.1371/journal.pone.0208780)
Supplement: S1 Table — (PDF) [file pone.0208780.s001.pdf]

Full names of the plant species mentioned in the paper

Clusters of high abundance of plants detected from local indicators of spatial association (LISA) in a semi-deciduous tropical forest

José Ramón Martínez Batlle[1*], Yntze van der Hoek[2,3]

1 Universidad Autónoma de Santo Domingo, Facultad de Ciencias, Ciudad Universitaria, Santo Domingo D.N., Dominican Republic, 2 Universidad Regional Amazónica Ikiam, Tena, Ecuador, 3 The Dian Fossey Gorilla Fund International, Musanze, Rwanda

***joseramon@geografiafisica.org**

Acacia skleroxyla Tussac
Amyris elemifera L.
Ateleia gummifera (Bertero ex DC.) D. Dietr.
Bursera simaruba (L.) Sarg.
Calliandra haematomma (Bertero ex DC.) Benth.
Chiococca alba (L.) Hitchc.
Chrysophyllum oliviforme L.
Coccoloba buchii O.C. Schmidt
Coccoloba diversifolia Jacq.
Coccothrinax argentea (Lodd. ex Schult. & Schult. f.) Sarg. ex K. Schum.
Comocladia dodonaea (L.) Urb.
Eugenia foetida Pers.
Eugenia odorata O. Berg
Exothea paniculata (Juss.) Radlk.
Guaiacum officinale L.
Guettarda mollis DC.
Jasminum fluminense Vell.
Leucaena leucocephala (Lam.) de Wit
Nectandra coriacea (Sw.) Griseb.
Ottoschulzia domingensis Urb.
Picramnia pentandra Sw.
Pictetia sulcata (P. Beauv.) Beyra & Lavin
Randia aculeata L.
Samyda dodecandra Jacq.
Savia sessiliflora (Sw.) Willd.
Swietenia mahagoni (L.) Jacq.
Tabebuia berteroi (DC.) Britton
Trichilia pallida Sw.
Vachellia macracantha (Humb. & Bonpl. ex Willd.) Seigler & Ebinger
